# Genome Sequence of a Multidrug-Resistant Strain of *Bacillus pumilus*, CB01, Isolated from the Feces of an American Crow, *Corvus brachyrhynchos*

**DOI:** 10.1128/genomeA.00807-16

**Published:** 2016-08-18

**Authors:** R. Lee Nelson, Michael A. Castro, Madhusudan Katti, Jonathan A. Eisen, Tricia A. Van Laar

**Affiliations:** a Department of Biology, College of Science and Mathematics, California State University, Fresno, Fresno, California, USA; b Department of Evolution and Ecology, Department of Medical Microbiology and Immunology, University of California, Davis, Davis, California, USA

## Abstract

Avian species have the potential to serve as important reservoirs for the spread of pathogenic microorganisms. Here, we report the genome sequence of a drug-resistant strain of *Bacillus pumilus*, CB01, isolated from the feces of an American crow, *Corvus brachyrhynchos*.

## GENOME ANNOUNCEMENT

Work in avian species, including gulls (1), crows (2), and other birds (3), has shown the potential for the transmission of pathogenic organisms to humans and human-associated settings, including poultry and dairy operations. More importantly, birds (4, 5) have been identified as being reservoirs for antimicrobial-resistant microorganisms. Birds, such as gulls and crows, are of particular concern as reservoirs for antimicrobial resistance due to their association with human habitation and tendency to feed on raw sewage and garbage (2, 3), which are likely contaminated with enteric pathogens and other pathogenic microorganisms. Also, birds are highly migratory, with approximately 300 species migrating to and from North America every year (3), leading to the potential spread of antimicrobial-resistant organisms to/from multiple countries.

*Bacillus pumilus* is a Gram-positive, aerobic, spore-forming bacterium found commonly in soil. The spores allow this organism to be resistant to many adverse environmental conditions, including heat, desiccation, and disinfection (6). *B. pumilus* has been shown to produce and secrete antifungal agents, promote plant growth, degrade pesticides, and control insect larvae (7–10). More recently, *B. pumilus* has also been shown to be associated with infection, including sepsis in neonates and cutaneous and catheter infections (11–14).

*B. pumilus* strain CB01 was isolated from the fecal matter from an American crow, *Corvus brachyrhynchos*, collected approximately 200 m from Fresno Community Regional Medical Center in Fresno, CA. Feces were resuspended in phosphate-buffered saline (PBS) and streaked for isolation on various differential media. This colony was selected from orange serum agar and grown in LB broth overnight at 37°C. Total genomic DNA was purified using the Wizard genomic DNA purification kit from Promega (Madison, WI, USA). This strain was identified as *B. pumilus* through 16S rRNA sequencing (15). As a major concern with bacterial infections is their potential for antimicrobial resistance, we determined the minimum inhibitory concentrations (MICs) of various antibiotics to generate a resistance profile for *B. pumilus* CB01. Our analysis found resistance to numerous antibiotics, including cefotaxime, hygromycin, methicillin, and spectinomycin.

*De novo* genome sequencing of *B. pumilus* CB01 was provided by MR DNA (Shallowater, TX) using an Illumina MiSeq, generating 3,217,896 300-bp paired-end reads. The sequence was processed and assembled using the A5-miseq assembly pipeline (16). Assembly yielded a genome with 3,830,520 bp and 41.5% G+C content, with a coverage of 169.89×. There were 37 contigs, with an *N*_50_ contig size of 442,844 bp. The genome was annotated using the RAST annotation server (17) and was predicted to contain 3,988 protein-coding sequences and 100 noncoding RNAs.

Continued study of microorganisms associated with avian species, particularly the American crow, will provide insight into the potential for these birds to contribute to pathogenesis and antimicrobial resistance in human populations.

### Accession number(s).

This whole-genome shotgun project has been deposited at DDBJ/ENA/GenBank under the accession no. LYXP00000000. The version described in this paper is version LYXP01000000.
